# Performance Analysis of Ten Common QRS Detectors on Different ECG Application Cases

**DOI:** 10.1155/2018/9050812

**Published:** 2018-05-08

**Authors:** Feifei Liu, Chengyu Liu, Xinge Jiang, Zhimin Zhang, Yatao Zhang, Jianqing Li, Shoushui Wei

**Affiliations:** ^1^The State Key Laboratory of Bioelectronics, Jiangsu Key Lab of Remote Measurement and Control, School of Instrument Science and Engineering, Southeast University, Nanjing, China; ^2^Shandong Zhong Yang Software Limited Company, Jinan, China; ^3^School of Control Science and Engineering, Shandong University, Jinan, China

## Abstract

A systematical evaluation work was performed on ten widely used and high-efficient QRS detection algorithms in this study, aiming at verifying their performances and usefulness in different application situations. Four experiments were carried on six internationally recognized databases. Firstly, in the test of high-quality ECG database versus low-quality ECG database, for high signal quality database, all ten QRS detection algorithms had very high detection accuracy (*F*1 >99%), whereas the *F*1 results decrease significantly for the poor signal-quality ECG signals (all <80%). Secondly, in the test of normal ECG database versus arrhythmic ECG database, all ten QRS detection algorithms had good *F*1 results for these two databases (all >95% except RS slope algorithm with 94.24% on normal ECG database and 94.44% on arrhythmia database). Thirdly, for the paced rhythm ECG database, all ten algorithms were immune to the paced beats (>94%) except the RS slope method, which only output a low *F*1 result of 78.99%. At last, the detection accuracies had obvious decreases when dealing with the dynamic telehealth ECG signals (all <80%) except OKB algorithm with 80.43%. Furthermore, the time costs from analyzing a 10 s ECG segment were given as the quantitative index of the computational complexity. All ten algorithms had high numerical efficiency (all <4 ms) except RS slope (94.07 ms) and sixth power algorithms (8.25 ms). And OKB algorithm had the highest numerical efficiency (1.54 ms).

## 1. Introduction

Cardiovascular diseases (CVDs) are the most common cause of death globally. In 2012, CVDs were the cause of death for about 17.5 million people, which equated to about 31% of all global deaths [1]. An electrocardiogram (ECG) signal, the expression of the myocardium electrical activity on the body's surface, provides important information about the status of cardiac activity [2]. The accurate and real-time heart beat detection of the ECG signal plays a fundamental role in monitoring of CVDs [3].

The QRS complex is the most striking waveform within the ECG signal. It serves as the basis for the automated determination of the heart rate, as well as the benchmark point for classifying the cardiac cycle and identifying any abnormality. Over the last few decades, the QRS complex detection has been extensively studied. In 1984, Pahlm and Sornmo discussed the QRS detection methods developed before 1984 in the aspects of digital preprocessing and detection rule, which is a very early paper for systematically analyzing the QRS detection methods [4]. In 2002, Köhler et al. reviewed and compared the great variety of QRS detection algorithms [5]. They grouped all the algorithms into four categories, respectively, based on signal derivatives, wavelet, neural network, and additional approaches. The algorithmic comparisons with respect to the computational load and detection accuracies were carried out to rate the algorithms. This literature was the most cited review paper about QRS detection algorithms. In 2014, Elgendi et al. investigated the existing QRS detection methodologies to target a universal fast-robust detector for portable, wearable, battery-operated, and wireless ECG systems [6]. This study compared the different QRS enhancement and detection techniques based on three assessment criteria: (1) robustness to noise, (2) parameter choice, and (3) numerical efficiency.

However, the review [4] did not compare the performances of different QRS detectors. In the review [5], the computational load and detection accuracies of QRS detection algorithms were not based on a standard database, and the comparison results were not given quantitatively. In the review [6], the comparison results were only based on the MIT-BIH arrhythmia database, but these results were from different literatures. In these literatures, some investigators have excluded some records [7] from the MIT-BIH arrhythmia database or excluded some segments with ventricular flutter [8] for the sake of reducing noise in the processed ECG signals.

In 1990, the noise sensitivities from nine different QRS detection algorithms were evaluated on a normal, single-channel lead, synthesized ECG database corrupted with five different types of synthesized noise [9]. In 2006, three methods were quantitatively compared using a similar algorithm structure but applying different transforms to the differentiated ECG [10]. The three transformations used were the Hilbert transformer, the squaring function, and a second discrete derivative stage. In 2008, the traditional first-derivative based squaring function method [11] and the Hilbert transform-based method [12], as well as their modifications with improved detection thresholds, were analyzed in the literature [13]. In 2013, Álvarez et al. analyzed the performances of three algorithms [14], Pan and Tompkins algorithm [15], Hamilton and Tompkins algorithm [11], and a phasor transform-based algorithm [16]. However, some studies [9, 10, 13, 14] quantitatively compared different QRS detection algorithms based on the same database, that is, the MIT-BIH arrhythmia database. The MIT-BIH arrhythmia database was widely used to evaluate QRS detection algorithms as it includes different shapes of arrhythmic QRS complexes and noise. As shown in many literatures, majority of the QRS detection algorithms had high detection sensitivity and positive predictivity on the MIT-BIH arrhythmia database (>99%) [1, 6]. However, performances of multiple algorithms on multiple source ECG databases lack. For example, the evaluation on ECG signals monitored by portable devices has not been systematically studied, which also challenges the current signal processing algorithms. The ECG signals recorded from the dynamic and mobile equipment are inevitably noise corrupted, consisting of more uncontrollable aspects, such as physiology, pathology, and artificial effects [17]. Therefore, the performance comparison of the commonly used algorithms should be extended to multiple source ECG databases.

In this study, the performances of ten widely used and high-efficient QRS detection algorithms were systematically evaluated on six ECG databases, with a special focus on the comparison between two opposite types or special application situations: high-quality ECG database versus low-quality ECG database, normal ECG database versus arrhythmic ECG database, paced rhythm ECG database, and dynamic telehealth ECG database. These ten algorithms were reported as high-efficient algorithms and suitable for mobile device situations [6, 17].

## 2. Methods

### 2.1. Databases

#### 2.1.1. High and Poor Signal Quality ECG Databases

Two hundred ECG records from the 2014 PhysioNet/CinC Challenge [12, 13] were used in this study. These records were from two databases: 100 records (named 100∼199, sampled at 250 Hz) from the training set and another 100 records (sampled at 360 Hz) from the augmented training set. Each record is 10 min long. The signal quality of ECG signals in the training set is always good, whereas the signal quality in the augmented training set is very poor. Thus, the training set was used as a high-quality ECG database and the augmented training set was used as a poor quality ECG database in this study.

#### 2.1.2. Normal Sinus Rhythm and Arrhythmia ECG Databases

Eighteen long-term ECG records from the MIT-BIH normal sinus rhythm (NSR) database were used as the normal subjects' data. Each record has a time length of two hours. ECG signals were sampled at 128 Hz. Subjects included in this database were found to have no significant arrhythmias. Besides, 44 of the 48 records from the MIT-BIH arrhythmia (ARR) database were used as the patients' data. Four records were excluded as they are paced ECGs. Each of the remaining 44 records had a time length of half an hour. ECG signals were sampled at 360 Hz.

#### 2.1.3. Pacemaker Rhythm ECG Database

Four records from the MIT-BIH arrhythmia database (records 102, 104, 107, and 217) including pacing signals were regarded as the pacemaker rhythm ECG database in this study.

#### 2.1.4. Telehealth ECG Database

Two hundred fifty lead-I ECGs from the TELE database [3] were used as telehealth ECG database in this study. These ECG records were recorded using the TeleMedCare Health Monitor (TeleMedCare Pty., Ltd., Sydney, Australia) in a telehealth environment [18] and were sampled at 500 Hz.

All ECG records from the above six databases selected in this study had manually annotated QRS complex locations, and these locations were used as the references for the algorithm evaluations [14].  Table 1 describes all these databases in detail.

### 2.2. Preprocessing

A unified signal preprocessing session was performed before QRS detection for the fair comparisons among different QRS detection methods. This session included three steps: flat line detection, signal detrending, and band-pass filtering.

#### 2.2.1. Flat Line Detection

ECG was detected as a flat line signal, if the portion of samples with constant amplitude was higher than 80% [19].

#### 2.2.2. Signal Detrending

Firstly, the least-squares fit of the ECG signal data was computed. Then, the best fitted value was removed from the ECG signal. The Matlab function “detrend.m” was used to remove the linear trend in the ECG signal.

#### 2.2.3. Band-Pass Filtering

The third-order Butterworth [20] band-pass filter was used to filter the ECG signal at a frequency range of 0.05–40 Hz. The Butterworth filter is a type of signal processing filter designed to have as flat a frequency response as possible in the passband. It is also referred to as a maximally flat magnitude filter.

### 2.3. QRS Detection Algorithms

As is known to all, QRS detection is a hot research topic for more than 40 years. A lot of QRS detectors have been proposed. It would be impractical to compare all of them. Three criteria for selecting the suitable algorithms were used in this study: algorithm efficiency, detection accuracy, and implementability. According to the three criteria, ten algorithms were selected from about 30 papers about QRS detection.

Any algorithm selected in this study should be widely used, with low computational complexity, and it could be executed in real-time circumstances on the mobile devices. As having limitations in terms of phone memory and processor capability, ECG monitoring using battery-operated, portable device is desirable for the efficient (fast and fewer calculations) QRS detection algorithms. Meanwhile, the QRS detection algorithms should have high detection accuracy, which is an essential basis for the actual applications. As we all know, researchers not always could write the right program according to the description of some papers. So, the implementability was also a key point for QRS detectors.

Table 2 shows the detailed information of these ten algorithms in four aspects. The first three methods were all Pan–Tompkins-based algorithms and based on the same principle, but there were many differences in the operating approach. For more information, see [21].

### 2.4. Evaluation Methods

The sensitivity (Se), positive predictivity (+*P*), and *F*1 measure [31] were used as the evaluation indexes, which are defined as follows:

(1) $$\begin{array}{c} \begin{array}{l} \mathrm{Se}=\frac{\mathrm{TP}}{\mathrm{TP}+\mathrm{FN}}\times 100\%,\\ +P=\frac{\mathrm{TP}}{\mathrm{TP}+\mathrm{FP}}\times 100\%,\\ F1=\frac{2\times \mathrm{TP}}{\left(2\times \mathrm{TP}+\mathrm{FP}+\mathrm{FN}\right)}\times 100\%, \end{array} \end{array}$$

where TP is the number of QRS complexes truly detected, FP is the number of false positive (extra falsely detected QRS complexes), and FN is the number of false negative (missed detected QRS complexes).

**Figure 1:**
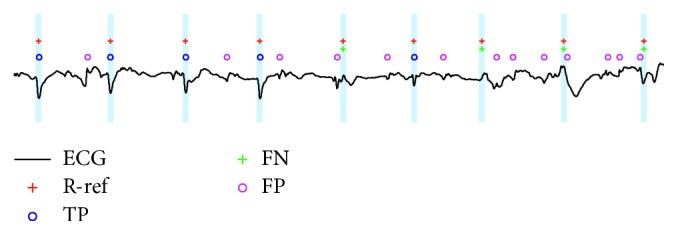
Example of TP (marked as blue “o”), FN (green “+”), and FP (pink “o”) detections from record 41,778 in the low-quality database. Reference QRS annotations (R-ref) are marked as red “+.” Vertical grey areas denote the tolerance time window of 50 ms.

Figure 1 shows an example of TP (marked as blue “o”), FN (green “+”), and FP (pink “o”) detections from the record 41,778 in the low-quality database. Red “+” signs indicated the reference QRS annotations (R-ref). A tolerance time window of 50 ms was used and denoted by the vertical grey areas to determine the TP detections. If the detected QRS location is within the current vertical grey area, it is considered as TP detection. If the detected QRS location is out of the current vertical grey area, it is considered as FP detection. If there is no detected QRS location within the current vertical grey area, it is considered to be FN detection. If more than one detected QRS locations exist within the current vertical grey area, one is considered to be TP detection and the others FP detection.

In this study, the ECG signal was firstly segmented into 10 s ECG episodes with a 50% overlap; that is, each episode had 5 s overlap with the previous one. Then the employed QRS detection algorithms were performed on each 10 s ECG episode. Then, the results of QRS locations from all 10 s episodes were integrated as the final algorithm output.

## 3. Results

**Figure 2:**
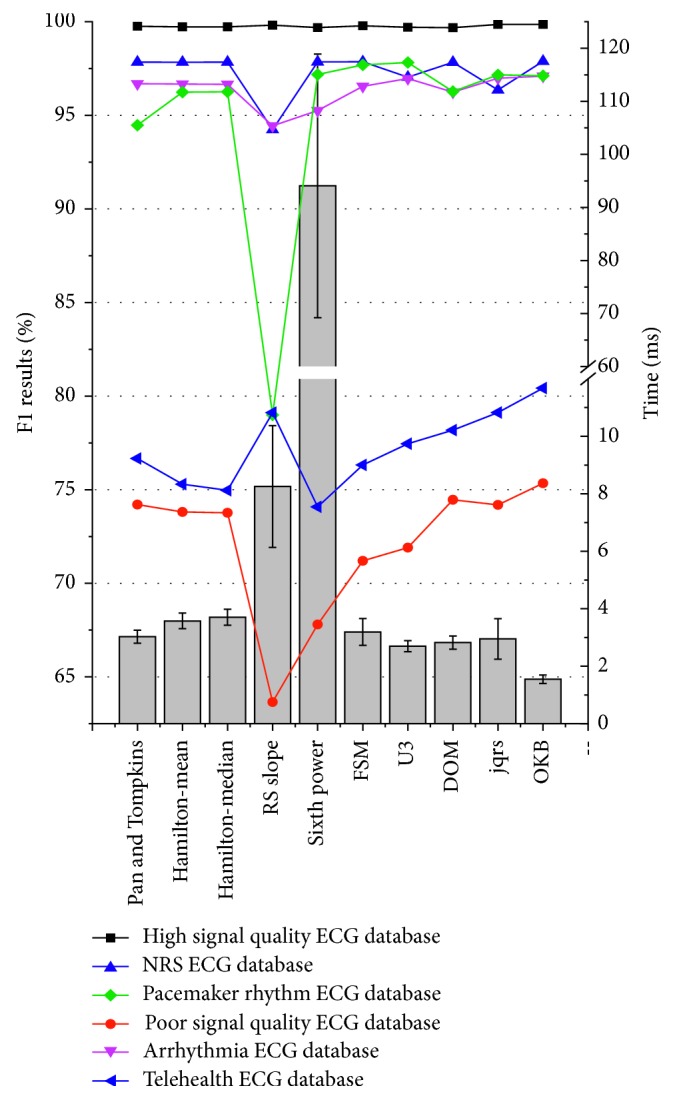
Line graph for *F*1 results and histogram for the average time costs.

Figure 2 illustrates the line graph for *F*1 results of the ten algorithms on these six ECG databases. All ten QRS detection algorithms had good *F*1 results for the high signal quality ECG data (all >99%, black square line). However, the *F*1 results decrease significantly for the poor signal quality ECG signals (all <80%, red round line), where the OKB algorithm reported the highest *F*1 result at 75.35%, while the RS slope algorithm gave the lowest *F*1 result of 63.66%. The blue equilateral triangle line and magenta inverted triangle line represent the results of the NSR and ARR ECG database, that is, the normal subjects and arrhythmia patients, respectively. All ten QRS detection algorithms had high *F*1 results for these two databases (all >95% except RS slope algorithm with 94.24% on NSR database and 94.44% on ARR database). The OKB algorithm still reported the highest *F*1 result of 97.89% and 97.09% on both databases. For the Paced-rhythm ECG database, all ten algorithms were immune to the paced beats (>94%) except the RS slope method, which only output a low *F*1 result of 78.99% (green rhombus line). However, for the telehealth database, the detection accuracies had obvious decline when dealing with the dynamic telehealth ECG signals. All the other nine algorithms reported *F*1 result lower than 80% except the OKB algorithm with an *F*1 score of 80.43%. Sixth power algorithm gave the lowest *F*1 result of 74.08% (black triangle line).

In this study, all of the tests were implemented in MATLAB 2014a (The MathWorks, Inc., Natick, MA, USA) on Intel TM i5 CPU 3.30 GHz. Figure 2 also illustrates the histogram for the time costs. This time costs were from analyzing an ECG segment (i.e., 10 s ECG signals in this study) on the six ECG databases. All ten algorithms had high numerical efficiency (all <4 ms) except RS slope (mean: 94.07 ms, SD: 24.85 ms) and sixth power algorithms (mean: 8.25 ms, SD: 2.12 ms). OKB algorithm had the highest numerical efficiency (mean: 1.54 ms, SD: 0.15 ms).

## 4. Discussion

In this study, the performances of ten widely used QRS detection algorithms with low computational complexity were systematically evaluated on six ECG databases, with a special focus on the comparison between two opposite types or special application situations: high-quality ECG database versus low-quality ECG database, normal ECG database versus arrhythmic ECG database, paced rhythm ECG database, and dynamic telehealth ECG database. These ten widely used algorithms were reported as very efficient algorithms and suitable for mobile device situations.

QRS detection has been extensively studied for over 40 years. However, most QRS detectors focused on clean clinical ECG data which are collected using gelled adhesive electrodes applied in precise locations. To the authors' best knowledge, a few of these detectors have been tested by ECG data with poor signal quality. In the literature [9], Gary et al. analyzed the performances of nine different QRS detection algorithms on the ECG data corrupted with five different types of synthesized calibrated noise and reported that the detection accuracies of these algorithms degraded with the noise level increasing. Xie et al. [32] and Khamis et al. [3] both reported that the performance of QRS detectors on the telehealth dynamic ECG database were poor if the detecting was carried without any preprocessing. The test results in this study also confirmed this case; that is, the detection accuracies of any detectors were not good for the ECG signal with poor signal quality and high noise level. How to settle this problem? In the literatures [3, 32], the artifact masking technology was used as a preprocessing step to avoid using noisy data in the calculation of means or thresholds during QRS detection. As reported, this technology highly improved the detection accuracies, but this did not remove the need for the QRS detector to be robust in the presence of some noise. In the PhysioNet/Computing in Cardiology Challenge 2014 [33], multimodal physiological signals were used to detect heart beats, which could improve the detection accuracy. In addition, the multilead ECG data fusion method [31, 34, 35] could be a promising method for QRS complex detection on the poor signal quality ECG database. In this paper, group A database included high and poor signal quality ECG databases. For the high signal quality ECG database, all ten QRS detection algorithms had high *F*1 (>99%), while the highest *F*1 result of poor signal quality database was only 75.35%.

ECG signals from different individuals show variability, and the variability is greater among healthy subjects and patients, especially for the patients with cardiac arrhythmia. Arrhythmia ECGs have different ECG patterns compared with the normal state. Different arrhythmia states, such as premature arrhythmias, ventricular arrhythmias, and conduction arrhythmias, present various ECG waveforms [37]. QRS detection is difficult because of the physiological variability of the QRS complexes. In addition, the irregular heart rate could increase the detection difficulty objectively [38]. However, the performances of ten algorithms tested in this paper did not decline significantly on the arrhythmias database. One possible reason was that the MIT-BIH arrhythmia database was widely used to evaluate QRS detection algorithms as it includes different shapes of arrhythmic QRS complexes and noise [3, 11, 15]. And some QRS detectors were optimized by this database [1]. In this study, all ten QRS detection algorithms had high *F*1 results for NSR and ARR databases (all >95% except RS slope algorithm with 94.24% on NSR database and 94.44% on ARR database). The OKB algorithm still reported the highest *F*1 result of 97.89% and 97.09% on both databases. In this algorithm, the optimized parameters were fixed through training on the MIT-BIH arrhythmia database using a rigorous brute-force search-based method.

**Figure 3:**
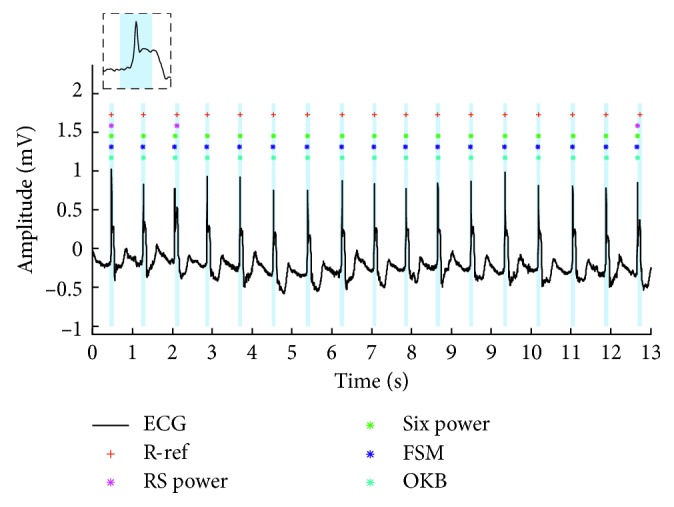
Example for the ventricular fusion beat.

The paced beat is another threat, especially for the algorithm based on slope and amplitude. However, in this study, only the performance of RS slope algorithm declined significantly unexpectedly. This algorithm distinguished the RS slope from other negative slopes based on the consistency of its amplitude and duration. In the paced ECG databases, there were many ventricular fusion beats including pacing irritation signal and QRS complex wave. The negative slope in the ventricular fusion beat was no longer prominent, as shown in Figure 3. In the ventricular fusion beat, this consistency had been destroyed. Because of that the number of false negative of RS slope algorithm was extremely big (RS slope algorithm: 3045 and the second largest was only 773). Other nine algorithms were robust to the effect of paced beat. Seven of these methods (Pan and Tompkins-based three algorithms [11, 15], FSM [25], U3 [26], “jqrs” [28], and OKB [1] algorithms) regarded peak energy as the characteristic value by integration, square or six power operations. The discontinuous RS slope has little influence on the peak energy extract. U3 transform algorithm used a nonlinear transform in the time-domain based on the curve-length concept [39], which was not influenced by the RS slope deformation. In the DOM algorithm [2], positive and negative threshold detection could remove this fluctuation in the RS slope.

The current advances in battery-driven devices such as smartphones and tablet computers have made these technologies a necessary part of daily life, even in developing countries [40]. In this way, the telehealth dynamic ECG database was used as an application test in this study. This database was collected by dry electrodes using the TeleMedCare health monitor. In this database, average 25.67% (SD 22.78) of each recording was visually identified as artifact, which was typical of data recorded in an unsupervised setting [3]. The literature [3] reported the detection results of three QRS detectors. When no special treatment was applied, the overall Se of the Pan and Tompkins [15] and FSM [25] algorithms was less than 50% and +*P* was less than 66%, whereas the UNSW algorithm [3] has an overall Se of 97.88% and +*P* of 71.67%. In this paper, the UNSW algorithm was not selected because of its high complexity. For this database, all other nine algorithms in this paper reported *F*1 result lower than 80% except that the OKB algorithm reported a *F*1 score of 80.43%. And sixth power algorithm gave the lowest *F*1 result of 74.08%.

With advances in computational power, the demand for numerical efficiency has decreased. However, this is still more the case when the ECG signals are collected and analyzed in hospitals, but not for the case of portable ECG devices, which are battery-driven [41]. Currently, portable battery-operated systems such as mobile phones with wireless ECG sensors have the potential to be used in continuous cardiac function assessment that can be easily integrated into daily life. However, there is a significant trade-off as there will always be a power-consumption limitation in processing ECG signals on battery-operated devices [42]. Recently, researchers have put an increased effort into developing efficient ECG analysis algorithms to run with mobile phones. Elgendi et al. [6] and Sufi et al. [17] both reported that the derivative and threshold are an efficient combination for detecting QRS if developed properly. They categorized the QRS detectors as low, medium, or high in terms of its numerical efficiency, based on the number of iterations and the number of equations employed, but not analyzed quantitatively. This study reported the time costs of these ten efficient QRS detectors as the quantitative index of the computational complexity. Although all these ten algorithms were based on the combination of derivative and threshold, the time costs were variable. Sixth power algorithm (mean: 94.07 ms, SD: 24.85 ms) was most time consuming because of the K point determination by the minima of the standard deviation of enhanced data with a fixed size of 16 samples. RS slope algorithm (mean: 8.25 ms, SD: 2.12 ms) was the second time-consuming algorithm due to ten groups of duration parameters detection. OKB algorithm (mean: 1.54 ms, SD: 0.15 ms) was the most efficient algorithm. The time cost of the other seven algorithms was about 3 ms.

There are some limitations in this study. Firstly, it should be noted that there must be many other good QRS detectors with high algorithm efficiency, detection accuracy, and operability. Due to the limited time and our viewpoints, only ten QRS detectors were selected in this study. Secondly, because some algorithms were published in a theoretical way without online code [1, 25] and some literatures only include a few guidelines for real implementation and do not fully explain the necessary preprocessing operations [23, 26], some QRS algorithms were coded by ourselves. Therefore, the detection results in this study may be different from those in the other literatures, but these differences are slight. Thirdly, a unified signal filtering was performed before QRS detection for the fair comparisons among different QRS detection methods. Then the second filtering operation was performed based on the different filtering requirements of different algorithms. However, the effect of the double-filtering was unknown. At last, for ECG database with poor signal quality, the performances of all these ten QRS detectors in this study were not good. How to improve the detection results on these databases with much noise will be a research focus.

We have carefully checked and verified the databases and algorithms employed in this paper and ensured the results' reliability. We are responsible for all the risks.

## 5. Conclusion

In this study, a systematical evaluation work was performed on ten widely used QRS detection algorithms with low computational complexity in different application situations.

Four experiments were carried on six internationally recognized databases. For the clean clinical ECG signals including normal subjects and arrhythmia patients, most QRS detectors have higher detection accuracies, whereas all these algorithms are not suitable for the poor signal quality ECG signals with high noise level. Thus, some special treatment methods need to be done for such case. For some special situation, such as paced rhythm, the QRS detector needs to be selected carefully. Although the derivative and threshold are an efficient combination for detecting the QRS complex wave, the preprocessing and postprocessing also have an influence on the computing cost. Therefore, the QRS detection algorithms need to be developed properly for the mobile ECG and portable battery-operated systems.

In conclusion, we have systematically evaluated ten widely used QRS detection algorithms and verified their performances and usefulness in different application situations. These results could offer reference for reasonably employing these algorithms.

## Figures and Tables

**Table 1:** The list of six databases.

Database	Description	Number of beats	Number of records	Record length (min)	Total time (min)	Sample frequency (Hz)	Source
A	High-quality ECGs	72,415	100	10	1000	250	2014 PhysioNet/CinC challenge training set (https://physionet.org/challenge/2014/)
	Low-quality ECGs	78,618	100	10	1000	360	2014 PhysioNet/CinC challenge augmented training set (https://physionet.org/challenge/2014/)
B	Normal subjects	1,806,792	18	120	2160	500	MIT-BIH NSR database (https://physionet.org/physiobank/database/nsrdb/)
	Arrhythmia patients	103,724	44	30	1320	360	MIT-BIH arrhythmia database (https://www.physionet.org/physiobank/database/mitdb/)
C	Paced rhythm ECGs	8923	4	30	120	360	MIT-BIH arrhythmia database (https://www.physionet.org/physiobank/database/mitdb/)
D	Telehealth environment ECGs	6708	250	0.5	125	500	Harvard dataverse TELE database (https://dataverse.harvard.edu/dataset.xhtml?persistentId=doi:10.7910/DVN/QTG0EP)
Total	—	**2,077,180**	**516**	—	**5725**	—	—

**Table 2:** Ten selected QRS detection algorithms.

Methods	Filtering	Extracting features	Setting threshold	Postprocessing
Pan–Tompkins algorithm [15]	5–15 Hz band-pass filter	Derivative; square; integrate	Two sets of adaptive thresholds	Searching back; T wave judging
Hamilton-mean algorithm [11]				
Hamilton-median algorithm [11]				
RS slope algorithm [21–23]	Median filter	Derivative; detecting negative slope	10 groups of duration empirical thresholds; one fixed amplitude threshold	200 ms refractory blanking technology
Sixth power algorithm [24]	Two-stage median filter	Sixth power	One adaptive threshold	Determining end point K
Finite state machine (FSM) algorithm [25]	/	Derivative; integrate; square	Three thresholding stages	/
U3 transform algorithm (U3) [26]	8–30 Hz band-pass filter	U3 transform	Two fixed thresholds	Searching back; 270 ms refractory blanking technology
Difference operation algorithm (DOM) [2, 27]	8–30 Hz band-pass filter	Derivative; detecting positive extreme points	Positive threshold; negative threshold	Optimizing; matching filtered signal
“jqrs” algorithm [28–30]	Sombrero hat-like low-pass filter	Integrate	One fixed threshold	Searching back; 200 ms refractory blanking technology
Optimized knowledge-based algorithm (OKB) [1]	8–20 Hz band-pass filter	Squaring; integration	Two dynamic thresholds	Determining the maxima of each block as R peak

## Data Availability

The data used to support the findings of this study are available from the corresponding author upon request.

## References

[1] Elgendi M. (2013). Fast QRS detection with an optimized knowledge-based method: evaluation on 11 standard ECG databases.

[2] Yeh Y. C., Wang W. J. (2008). QRS complexes detection for ECG signal: the difference operation method.

[3] Khamis H., Weiss R., Xie Y., Chen C. W., Lovell N., Redmond S. (2016). QRS detection algorithm for telehealth electrocardiogram recordings.

[4] Pahlm O., Sörnmo L. (1984). Software QRS detection in ambulatory monitoring—a review.

[5] Kohler B. U., Hennig C., Orglmeister R. (2002). The principles of software QRS detection.

[6] Elgendi M., Eskofier B., Dokos S., Abbott D. (2014). Revisiting QRS detection methodologies for portable, wearable, battery-operated, and wireless ECG systems.

[7] Moody G. B., Mark R. G. (2001). The impact of the MIT-BIH arrhythmia database.

[8] Martinez J. P., Almeida R., Olmos S., Rocha A. P. (2004). A wavelet-based ECG delineator: evaluation on standard databases.

[9] Friesen G. M., Jannett T. C., Jadallah M. A., Yates S. L., Quint S. R., Nagle H. T. (1990). A comparison of the noise sensitivity of nine QRS detection algorithms.

[10] Arzeno N. M., Poon C. S., Deng Z. D. Quantitative analysis of QRS detection algorithms based on the first derivative of the ECG.

[11] Hamilton P. S., Tompkins W. J. (1986). Quantitative investigation of QRS detection rules using the MIT/BIH arrhythmia database.

[12] Benitez D. S., Gaydecki P. A., Zaidi A., Fitzpatrick A. P. A new QRS detection algorithm based on the Hilbert transform.

[13] Arzeno N. M., Deng Z. D., Poon C. S. (2008). Analysis of first-derivative based QRS detection algorithms.

[14] Álvarez R. A., Penín A. J. M., Sobrino X. A. V. (2013). A comparison of three QRS detection algorithms over a public database.

[15] Pan J., Tompkins W. J. (1985). A real-time QRS detection algorithm.

[16] Martínez A., Alcaraz R., Rieta J. J. (2010). Application of the phasor transform for automatic delineation of single-lead ECG fiducial points.

[17] Sufi F., Fang Q., Cosic I. ECG R-R peak detection on mobile phones.

[18] Redmond S. J., Xie Y., Chang D., Basilakis J., Lovell N. H. (2012). Electrocardiogram signal quality measures for unsupervised telehealth environments.

[19] Hayn D., Jammerbund B., Schreier G. (2012). QRS detection based ECG quality assessment.

[20] Bianchi G. (2007).

[21] Podziemski P., Gieraltowski J. Fetal heart rate discovery: algorithm for detection of fetal heart rate from noisy, noninvasive fetal ECG recordings.

[22] Gieraltowski J. J., Ciuchcinski K., Grzegorczyk I., Kosna K. Heart rate variability discovery: algorithm for detection of heart rate from noisy, multimodal recordings.

[23] Gieraltowski J., Ciuchcinski K., Grzegorczyk I., Kosna K., Solinski M., Podziemski P. (2015). RS slope detection algorithm for extraction of heart rate from noisy, multimodal recordings.

[24] Dohare A. K., Kumar V., Kumar R. (2013). An efficient new method for the detection of QRS in electrocardiogram.

[25] Gutierrez-Rivas R., Garcia J., Marnane W., Hernandez A. (2015). Novel real-time low-complexity QRS complex detector based on adaptive thresholding.

[26] Paoletti M., Marchesi C. (2006). Discovering dangerous patterns in long-term ambulatory ECG recordings using a fast QRS detection algorithm and explorative data analysis.

[27] Cooman T. D., Goovaerts G., Varon C., Widjaja D., Willemen T., Huffel S. V. (2015). Heart beat detection in multimodal data using automatic relevant signal detection.

[28] Behar J., Oster J., Clifford G. D. Non-invasive FECG extraction from a set of abdominal sensors.

[29] Behar J., Oster J., Clifford G. D. (2014). Combining and benchmarking methods of foetal ECG extraction without maternal or scalp electrode data.

[30] Johnson A. E., Behar J., Andreotti F., Clifford G. D., Oster J. (2015). Multimodal heart beat detection using signal quality indices.

[31] Laguna P., Jan R., Caminal P. (1994). Automatic detection of wave boundaries in multilead ECG signals: validation with the CSE database.

[32] Xie Y., Redmond S. J., Basilakis J., Lovell N. H. Effect of ECG quality measures on piecewise-linear trend detection for telehealth decision support systems.

[33] Moody G., Moody B., Silva I. Robust detection of heart beats in multimodal data.

[34] Ledezma C. A., Perpiñan G., Severeyn E., Altuve M. Data fusion for QRS complex detection in multi-lead electrocardiogram recordings.

[35] Torbey S., Akl S. G., Redfearn D. P. Multi-lead QRS detection using window pairs.

[36] Llamedo Soria M., Martinez J. P., Laguna P. A multilead wavelet-based ECG delineator based on the RMS signal.

[37] Chang K. M. (2010). Arrhythmia ECG noise reduction by ensemble empirical mode decomposition.

[38] Sushma N., Sunil T. D., Kurian M. Z. (2015). Implementation of QRS peak detector by morphological operation and ECG extraction method for arrhythmia detection.

[39] Paoletti M., Marchesi C. Model based signal characterisation for long-term personal monitoring.

[40] Silva I., Moody G. B., Celi L. Improving the quality of ECGs collected using mobile phones.

[41] Kim H., Yazicioglu R. F., Merken P., Van H. C., Yoo H. J. (2010). ECG signal compression and classification algorithm with quad level vector for ECG Holter system.

[42] Hii P. C., Chung W. Y. (2011). A comprehensive ubiquitous healthcare solution on an Android™ mobile device.

